# Esrrb Is a Pivotal Target of the Gsk3/Tcf3 Axis Regulating Embryonic Stem Cell Self-Renewal

**DOI:** 10.1016/j.stem.2013.04.020

**Published:** 2013-05-02

**Authors:** Graziano Martello, Toshimi Sugimoto, Evangelia Diamanti, Anagha Joshi, Rebecca Hannah, Satoshi Ohtsuka, Berthold Göttgens, Hitoshi Niwa, Austin Smith

(Cell Stem Cell *11*, 491–504; October 4, 2012)

In this paper we erroneously stated on page 494: “In bulk culture in the presence of PD alone, PB-vector and PBTcfcp2l1 cells collapsed within two passages. Other transfectants could be maintained longer but progressively lost Rex1GFP expression (Figure 3B) and completely differentiated or died by five passages.” In fact we did not include Tcfcp2l1 transfectants in that particular experiment. The text should say: “In bulk culture in the presence of PD alone, PB-vector cells collapsed within two passages. Nr0B1, Nanog, or Klf2 transfectants could be maintained longer but progressively lost Rex1GFP expression (Figure 3B) and completely differentiated or died by five passages.” We apologize for any confusion caused.

